# Rapid Assessment of Insect Steroid Hormone Entry Into Cultured Cells

**DOI:** 10.3389/fphys.2021.816058

**Published:** 2022-01-26

**Authors:** Mitchell Masterson, Riyan Bittar, Hannah Chu, Naoki Yamanaka, Sachiko Haga-Yamanaka

**Affiliations:** ^1^Department of Molecular, Cell and Systems Biology, University of California, Riverside, Riverside, CA, United States; ^2^Department of Entomology, University of California, Riverside, Riverside, CA, United States

**Keywords:** ecdysteroid, transporter, cellular uptake, steroid hormone, Ecdysone Importer, NanoBiT assay, ecdysone receptor (EcR), nuclear receptor (NR)

## Abstract

Steroid hormones control development and homeostasis in a wide variety of animals by interacting with intracellular nuclear receptors. Recent discoveries in the fruit fly *Drosophila melanogaster* revealed that insect steroid hormones or ecdysteroids are incorporated into cells through a membrane transporter named Ecdysone Importer (EcI), which may become a novel target for manipulating steroid hormone signaling in insects. In this study, we established an assay system that can rapidly assess EcI-mediated ecdysteroid entry into cultured cells. Using NanoLuc Binary Technology (NanoBiT), we first developed an assay to detect ligand-dependent heterodimerization of the ecdysone receptor (EcR) and retinoid X receptor (RXR) in human embryonic kidney (HEK) 293T cells. We also developed HEK293 cells that stably express EcI. By combining these tools, we can monitor ecdysteroid entry into the cells in real time, making it a reliable system to assess EcI-mediated steroid hormone incorporation into animal cells.

## Introduction

Steroid hormones are cholesterol derivatives essential for development and homeostasis in a wide variety of animals. They not only regulate physiological processes including immune response and sexual maturation (Sapolsky et al., 2000; Sisk and Foster, 2004; Rhen and Cidlowski, 2005; Wilson and Davies, 2007; Oakley and Cidlowski, 2011; Sakiani et al., 2013), but also affect pathological processes such as cancer progression (Clemons and Goss, 2001; Rhen and Cidlowski, 2005; Attard et al., 2009). Disruption to steroid hormone signaling can therefore cause detrimental effects, while components of the signaling pathway can provide potential molecular targets for the treatment of various pathological conditions.

Steroid hormones are delivered *via* the circulatory system to target cells, where they bind to intracellular receptors called nuclear receptors (Mangelsdorf et al., 1995; Nilsson et al., 2001; King-Jones and Thummel, 2005; Evans and Mangelsdorf, 2014). Activated nuclear receptors in turn direct expression of a series of genes and alter cellular functions (McKenna and O’Malley, 2002). In arthropods, ecdysteroids are the class of steroid hormones that regulate development through controlling molting and metamorphosis (Riddiford et al., 2000; Hill et al., 2013; Truman, 2019; Yamanaka, 2021). Ecdysteroid actions are mediated by intracellular nuclear receptors, the ecdysone receptor (EcR) and Ultraspiracle (USP; Yao et al., 1992, 1993; Thomas et al., 1993). Upon ligand binding, EcR forms a heterodimeric structure with USP, the homolog of the mammalian retinoid X receptors (RXRs) in insects. The EcR/USP heterodimer complex then binds to the ecdysone response element (EcRE) on the genomic DNA and acts as a transcriptional regulator (Yao et al., 1992, 1993; Thomas et al., 1993).

Ecdysone receptor has been utilized as a target for a group of insecticides called insect growth regulators (IGRs; Dhadialla et al., 2005; Pener and Dhadialla, 2012; Okamoto and Yamanaka, 2021). For example, diacylhydrazine (DAH)-based non-steroidal ecdysone agonists, such as chromafenozide (CF), activate EcR, disrupt developmental transition, and eventually kill the insects (Wing et al., 1988; Nakagawa and Henrich, 2009; Smagghe et al., 2012). However, resistance to DAH-based ecdysone agonists has been recently reported in field populations of several insect species (Smagghe et al., 2012). This is partly because these IGRs need to be taken up to the cells to interact with their target protein, EcR, and therefore they readily become targets for intracellular detoxification enzymes such as cytochrome P450 monooxygenases, as well as multidrug ABC transporters that eliminate various pesticides out of the cells (Smagghe et al., 1998, 2001; Hock et al., 2000; Retnakaran et al., 2001; Uchibori-Asano et al., 2019).

Recently, a series of studies conducted in the fruit fly *Drosophila melanogaster* revealed that membrane transporters have critical functions in trafficking ecdysteroids across cell membranes (Yamanaka et al., 2015; Okamoto et al., 2018; Okamoto and Yamanaka, 2020). *Ecdysone Importer* (*EcI*) is a member of the solute carrier organic anion transporter gene superfamily that encodes organic anion transporting polypeptide 74D (OATP74D; Okamoto et al., 2018). Both *in vivo* and *in vitro* studies demonstrated that *EcI* is necessary and sufficient for cellular uptake of ecdysteroids (Okamoto et al., 2018; Okamoto and Yamanaka, 2020). As plasma membrane transporters are readily accessible from the extracellular space, such membrane steroid hormone importers may become alternative targets for chemical reagents that modulate steroid hormone signaling.

To identify chemical reagents that target steroid hormone importers, it is crucial to establish an assay system that can specifically detect cellular steroid hormone uptake with high sensitivity. In a previous study, a traditional luciferase reporter assay was conducted using human embryonic kidney (HEK) 293 cells, in which luciferase reporter activity was measured after 24-h treatment of the active ecdysteroid, 20-hydroxyecdysone (20E). While this assay successfully demonstrated sufficiency of EcI for cellular uptake of ecdysteroids, its relatively low sensitivity and long incubation time may cause various unwanted side effects in a drug screening process. Alternatively, direct measurements of the amount of intracellular 20E using radioisotopes or enzyme-linked immunoassay are possible ways to assess incorporation of 20E into the cells. However, these experiments are not trivial partly because leakage of 20E out of the cells needs to be carefully prevented throughout the assays.

In this study, in order to develop a more rapid and sensitive assay system for cellular uptake of ecdysteroids, we took advantage of NanoLuc Binary Technology (NanoBiT) to detect the formation of the EcR heterodimer complex. NanoBiT allows quantification of protein-protein interactions by utilizing a split luciferase-based tool that luminesces when the two proteins are in close contact (Dixon et al., 2016). We first optimized the assay system in HEK293T cells, relying on EcI introduction *via* transfection, and then established HEK293 cells which stably express EcI. This provides a simplified system sensitive to ecdysteroid entry into the cell through EcI while excluding additional biological processes of gene transcription and translation required for the classical luciferase assay. These tools will be useful for identifying chemical reagents that potentially either inhibit or facilitate cellular uptake of 20E through EcI.

## Materials and Methods

### Cloning NanoLuc Binary Technology Vectors

All NanoBiT expression vectors for mammalian expression were prepared using the backbones provided with the NanoBiT PPI Flexi Starter Kit (Promega, N2015). *EcR* and *USP* cDNA clones were obtained from *Drosophila* Genomics Resource Center (DGRC, RE33854, and LD09973, respectively), and *RXR* cDNA clone was purchased from Promega’s Kazusa collection (pFN21AB9770). In short, the *EcR* and *USP* sequences were amplified using primers which introduced *Sgf*I/*Asi*SI and *Pme*I restriction sites (primer sequences are provided in Table 1). One base was included between the *Asi*SI restriction site and the start codon, to maintain the reading frame for the N-terminal fusions, and the stop codons were not included, so that the C-terminal fusions would be expressed, stop codons were included in all plasmid types. The amplified products were agarose gel purified and digested using *Asi*SI (NEB, R0630S) and *Pme*I (NEB, R0560S). The four NanoBiT Flexi vectors were similarly digested with *Asi*SI and *Pme*I (for the N-terminal fusion plasmids) or *Eco*53kI (for the C-terminal fusion plasmids, NEB, R0116S) and dephosphorylated by Calf Intestinal Alkaline Phosphatase (NEB, M0290) in the same reaction before agarose gel purification. Using T4 DNA ligase (NEB, M0202S), the digested *EcR* and *USP* genes were ligated into the pFN33K N-terminal LgBiT TK-neo Flexi Vector with kanamycin resistance. The ligation was used in bacterial transformation with kanamycin selection to produce colonies with the desired inserted vectors, confirmed by sequencing. The produced pFN33K vectors were used to produce inserts for the other three NanoBiT plasmids for each gene by digestion with *Asi*SI and *Pme*I and gel purification of the *EcR* and *USP* inserts. The use of the plasmid as the source of the gene sequence once the first plasmid was cloned was a more efficient method of production of the digested sequence than the PCR and then digestion. These inserts were then ligated into each of the remaining NanoBiT plasmids for each gene; pFC34K C-terminal LgBiT TK-neo, pFC36K C-terminal SmBiT TK-neo, and pFN35K N-terminal SmBiT TK-neo Flexi Vectors all with kanamycin resistance. The ligation mix was used in bacterial transformation with kanamycin selection to produce colonies with the desired NanoBiT vectors. All the constructs are presented as a schematic representation in Supplementary Figure 1. *RXR* was purchased in a Flexi cDNA construct so was directly digested out by use of *Asi*SI and *Pme*I and the purified insert ligated into the NanoBiT vectors as above without the need for PCR introduction of the restriction sites. All vectors were sequenced to ensure that the reading frame was intact.

**TABLE 1:** Primer sequences for NanoBiT cloning.

Primer	Sequence (5′–3′)
EcR-*Asi*SI-Forward	ATTGCGATCGCCATGAAGCGGCGCTGGTCG
EcR-*Pme*I-Reverse	TTTGTTTAAACTGCAGTCGTCGAGTGCTCC
USP-*Asi*SI -Forward	ATTGCGATCGCCATGGACAACTGCGACCAGG
USP-*Pme*I-Reverse	TTTGTTTAAACCTCCAGTTTCATCGCCAGG

*Restriction sites are indicated with underlines.*

### Cell Culture

HEK293T cells were cultured in DMEM with 4.5 g/L glucose, L-glutamine, and sodium pyruvate (Thermo Fisher Scientific, 10-013-CV) with 10% FBS (Gibco, 10437028), and 1% penicillin-streptomycin (Gibco, 15140122). Custom-made HEK293 cells stably expressing *EcI* (HEK293-EcI) under a CMV promoter were generated by Thermo Fisher Scientific using Jump-In™ GripTite™ HEK293 cells (HEK293-Ctrl). These cells were cultured in DMEM with 4.5 g/L glucose, L-glutamine, and sodium pyruvate with 10% Heat Inactivated (HI) FBS (Gibco, 10082147) and 1% penicillin-streptomycin. Both cell types were cultured at 37°C in a 5% CO_2_ incubator. Prior to each experiment, cells were counted by using Bio-Rad TC20 cell counter.

### HEK293T Cell Transfection for the NanoLuc Binary Technology Assay

HEK293T cells were transfected 1–3 days after plating in DMEM with 4.5 g/L glucose, L-glutamine, and sodium pyruvate with 10% charcoal stripped FBS (Corning, MT35072CV) at 60–80% confluency with culture medium refreshed immediately prior. Transfection was performed using Attractene transfection reagent (Qiagen, 301005) according to the manufacturer’s instructions after optimization of the DNA: Attractene ratio. For 6 cm plates, a 150 μL transfection cocktail consisted of 0.5 μg of each NanoBiT plasmid for the pair (outside of partner testing experiments this was specifically pFC34K-*EcR* and pFC36K-*RXR* aka EcR-LgBiT and RXR-SmBiT), 1 μg pcDNA3.1-*EcI* or empty pcDNA3.1 and 7.5 μL Attractene in Opti-MEM. This was scaled as appropriate for other plate sizes when differing amounts of cells per transfection were required. After addition of the transfection reagents cells were incubated at 37°C and 5% CO_2_ overnight before seeding into 96-well plates.

### HEK293-Ecdysone Importer or HEK293-Ctrl Cell Transfection for the NanoLuc Binary Technology Assay

HEK293-EcI and -Ctrl cells were transfected 1–3 days after plating in DMEM with 4.5 g/L glucose, L-glutamine, and sodium pyruvate with 10% HI FBS and 1% penicillin-streptomycin at 70–90% confluency. The culture medium was refreshed immediately prior to transfection. Transfection was performed using Lipofectamine 3000 (Invitrogen, L3000008) according to the manufacturer’s instructions after optimization of DNA: lipofectamine ratio. For a 6-well plate, 250 μL transfection cocktail consisted of 1.25 μg of each pFC34K-*EcR* and pFC36K-*RXR*, 5 μL P3000 and 6 μL lipofectamine reagent in Opti-MEM. After addition of the transfection reagents, cells were incubated at 37°C and 5% CO_2_ overnight before seeding into 96-well plates.

### NanoLuc Binary Technology Assay

Transfected cells were seeded in white 96-well plates at a density of 4.5 × 10^4^ cells per well in 100 μL of DMEM with 10% charcoal stripped FBS. Following the NanoBiT protocol all outer wells were filled with 150 μL of water and the space between wells filled with 75 μL water to keep temperature close to 37°C during live cell assay. The plates were incubated at 37°C and 5% CO_2_ for 24 h before the assay. The culture medium was then aspirated and replaced with 100 μL DMEM without any additives and without phenol red. Nano-Glo live cell reagent (Promega, N2013) was prepared according to Promega’s guidelines, and 25 μL was added to each well followed by mixing the plates for 15 s. Luminescence was read every minute for 17 min using a VICTOR X3 luminometer (Perkin Elmer), set at 37°C. Treatments [20E (Sigma-Aldrich, H5142), CF (MedChem Express, HY-17533), or ethanol controls prepared in DMEM without phenol red] were then added into the wells in a 10 μL volume, and luminescence was read every minute for 45 more minutes.

### Handling Luminescence Data

To obtain relative luminescence, post-treatment luminescence reads for each well were normalized by division of the recorded luminescence by the average luminescence of the 4 measurements recorded immediately prior to treatment during the equilibration phase. Among the relative luminescence values within the 45-min post 20E/CF/EtOH application, the peak value was identified as the maximum luminescence for each replicate individually.

### Immunocytochemistry

HEK293-Ctrl and -EcI cells were seeded onto poly-D-lysine hydrobromide (Sigma-Aldrich) coated coverslips in 24-well plates. After overnight culture the cells were fixed in 4% paraformaldehyde (Thermo Fisher Scientific, 41678-5000) in PBS for 10 min at 37°C and then washed three times with PBS. The fixed cells were permeabilized by incubation in 0.5% Triton X-100 (Sigma-Aldrich) in PBS for 5 min at room temperature while shaking and washed three more times with PBS. The cells were blocked by incubation while shaking for 1 h 30 min in blocking buffer (5% bovine serum albumin, 0.1% Tween 20 in PBS) and incubated at 4°C overnight with anti-EcI primary antibody [generated and affinity purified by Pierce Biotechnology, Inc. (Okamoto and Yamanaka, 2020)] at 1:50 in blocking buffer. The cells were washed 3 times with 0.1% Triton X-100 in PBS (PBST) and incubated for 1 h with Alexa Fluor 488-conjugated goat anti-guinea pig IgG secondary antibody (Thermo Fisher Scientific, A11073) at 1:500 in blocking buffer before being washed three more times in PBST. The cells were then exposed to DyLight™ 554 phalloidin (Cell Signaling Technologies, 13054) at 1:200 in PBS for 15 min at room temperature to stain the cytoskeleton. The cells were washed three times with PBS, mounted in EMS Shield Mounting Medium with DAPI and DABCO™ (EMS, 17989-20), and observed using a 63× objective lens with Zeiss Axio Imager M2 equipped with ApoTome.2.

### Ecdysteroid-Inducible Luciferase Reporter Assay in HEK293-Ecdysone Importer Cells

HEK293-EcI cells were transfected 2 days after seeding at a density of 3 × 10^5^ cells/mL in 6-well plates in DMEM with 4.5 g/L glucose, L-glutamine, and sodium pyruvate with 10% HI FBS, and 1% penicillin-streptomycin. The culture medium was refreshed on the day of transfection. Transfection was performed using Lipofectamine 3000 following the manufacturer’s instructions. For all experiments, 1.5 μg/well of *pERV3* receptor plasmid (Agilent Technologies, 217468) was transfected along with 750 ng/well of *pEGSH-LUC* luciferase reporter plasmid (Agilent Technologies, 217468), and 250 ng/well of *pRL-CMV* Renilla luciferase reporter plasmid (Promega, E2261) as a reference. After 2 days of incubation in the conditions described above, the cells were washed twice with PBS and then transferred to a clear 96-well plate in DMEM with 4.5 g/L glucose, sodium pyruvate; without L-glutamine, phenol red at 100 μL/well. Final concentrations ranging from 300 to 300 μM of 20E or CF were added immediately after, with a final volume of 125 μL/well. The treated cells were then incubated for 1 day in the same conditions. The Dual-Luciferase Reporter Assay was performed as described previously (Okamoto et al., 2018).

## Results

### Optimization of the NanoLuc Binary Technology Assay to Detect 20-Hydroxyecdysone Entry Into HEK293T Cells

**FIGURE 1:**
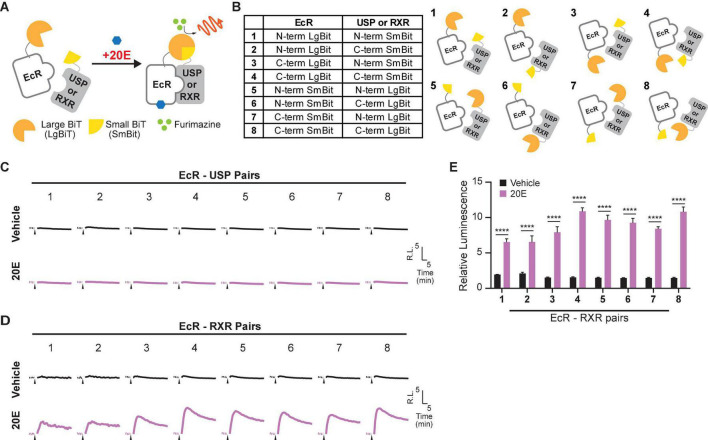
Optimization of the EcR-RXR NanoBiT assay. **(A)** Schematic diagram of the NanoBiT 20E reporter system. EcR and either USP or RXR are expressed as fusion proteins with LgBiT and SmBiT, respectively. When the NanoBiT partners are brought in close proximity by EcR dimerization with USP or RXR in the presence of 20E, the NanoBiT partners form a functional luciferase enzyme which produces an observable light signal in the presence of the substrate furimazine. **(B)** Table (left) and schematics (right) showing the fusion protein pairs used in optimization testing. Eight different options are available as LgBiT or SmBiT could be fused to either protein and the fusion at either the N- or C-terminus. **(C,D)** Mean time-course changes of the relative luminescence in HEK293T cells expressing EcI and each of the NanoBiT EcR-USP **(C)** or EcR-RXR **(D)** combinations 1–8 listed in panel **(B)**. Black triangles indicate the time point when either EtOH or 10 μM 20E was added to the medium. Data were normalized to the average of the basal luminescence reading immediately prior to treatment (dashed lines). R.L., relative luminescence. **(E)** Maximum relative luminescence from the EcR-RXR pairs for each of the combinations 1–8 listed in panel **(B)**. Bars represent means ± S.E.M. (*n* = 3). Analysis by two-way ANOVA identified a significant interaction of the NanoBiT pair in response to the 20E treatment [*F*_(7, 32)_ = 8.32, *p* < 0.0001]. ****p < 0.0001 by multiple comparisons using Sidak’s correction.

NanoLuc Binary Technology is a binary assay system based on two luciferase subunits: the large subunit, (LgBiT; 17.6 kDa) and the small subunit (SmBiT; 11 amino acids). LgBiT and SmBiT can be fused to two interacting target proteins, such as the nuclear receptors EcR and USP. When these proteins interact, the two subunits are brought into proximity, which allows structural complementation to form a functional luciferase enzyme (Figure 1A). LgBiT and SmBiT can be fused to either N- or C-terminus of target proteins, as long as those subunits can interact with each other without interfering the target protein interaction. Therefore, finding the best terminus-subunit combination to tag each target protein is the first step to establish this assay system.

To find the optimal pair for detecting EcR signaling, we first generated expression plasmids for EcR and USP fused with LgBiT or SmBiT at either the N- or C-terminus: four EcR plasmids (N-terminus LgBiT, C-terminus LgBiT, N-terminus SmBiT, and C-terminus SmBiT) and four USP plasmids (N-terminus LgBiT, C-terminus LgBiT, N-terminus SmBiT, and C-terminus SmBiT). Plasmid pairs carrying eight possible protein tagging combinations of EcR and USP (Figure 1B) were then transfected into HEK293T cells together with an *EcI*-containing plasmid. The real-time luminescence response to vehicle or 10 μM 20E was monitored after measuring background luminescence. Unexpectedly, none of the EcR-USP pairs exhibited increased luminescence in response to 20E. Similar to the vehicle control, the luminescence levels after 20E application did not change from pre-application baselines (Figure 1C).

Ecdysone receptor can also form a heterodimer with the mammalian homolog of USP, RXR (Yao et al., 1992, 1993; Thomas et al., 1993). Indeed, RXR has been used as an EcR binding partner for some ecdysone-inducible gene switches in *in vitro* and *in vivo* mammalian models (No et al., 1996; Saez et al., 2000; Wyborski et al., 2001; Palli et al., 2005). Thus, we next generated four RXR NanoBiT plasmids (N-terminus LgBiT, C-terminus LgBiT, N-terminus SmBiT, and C-terminus SmBiT) and transfected them with *EcR*- and *EcI*-containing plasmids into HEK293T cells. We found that all eight combinations of EcR-RXR pairs (Figure 1B) induced luminescence increase after 20E application, while vehicle treatment did not (Figure 1D). The levels of relative luminescence upon 20E treatment were different among the EcR-RXR pairs (Figure 1E), with 6 to 10 times greater relative luminescence at the maximum level after 20E application. These levels were significantly higher when compared to the vehicle control for each NanoBiT combination (all *p* < 0.0001, *n* = 3) (Devarakonda et al., 2003).

Taken together, we conclude that EcR and RXR are a better protein pair for detecting 20E-dependent receptor heterodimerization using NanoBiT in HEK293T cells compared to EcR and USP. We also note that LgBiT and SmBiT show optimal response when fused at C-termini of EcR and RXR proteins. The combination of EcR with a C-terminal LgBiT (EcR-LgBiT) and RXR with a C-terminal SmBiT (RXR-SmBiT) was used for all the following experiments (pair 4). Of the two C-terminal fusion combinations (pairs 4 and 8), this pair was chosen due to slightly lower background (Supplementary Figure 2), although no substantial difference in relative response between these two pairs was observed.

### Response of the NanoLuc Binary Technology Luciferase Reporter to 20-Hydroxyecdysone and Chromafenozide

Dose-dependent EcR response to 20E was observed in a previous study using an ecdysteroid-inducible luciferase reporter assay in *EcI*-expressing HEK293T cells (Okamoto et al., 2018). EcR activation in *EcI*-expressing cells was significantly induced by 20E concentrations of at least 3 μM.

**FIGURE 2:**
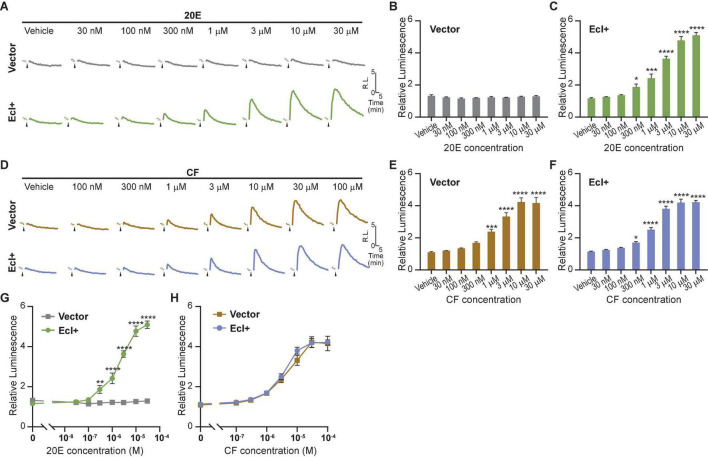
Detection of 20E and CF entry into HEK293T cells using the NanoBiT assay. **(A)** Mean time-course changes of the relative luminescence in HEK293T cells transfected with RXR-SmBiT and EcR-LgBiT reporters. Either an *EcI*-containing vector (EcI +) or an empty vector (Vector) was co-transfected, and the luminescence was monitored in real time in response to different doses of 20E. Black triangles indicate the time point when 20E was added to the medium. Data were normalized to the average of the basal luminescence reading immediately prior to treatment (dashed lines). R.L., relative luminescence. **(B,C)** Maximum relative luminescence in response to different doses of 20E in the Vector **(B)** or EcI + **(C)** HEK293T cells. Bars represent means ± S.E.M. (*n* = 4). One-way ANOVA analysis identified no significant effect in the Vector cells [*F*_(7,24)_ = 1.16, *p* = 0.362, **(B)**], whereas significant effects were observed in the EcI + cells [*F*_(7,24)_ = 85.97, *p* < 0.0001, **(C)**]. *, ***, and **** indicate *p* < 0.05, 0.001, and 0.0001, respectively, from multiple comparisons to the vehicle treatment using Dunnett’s correction. **(D)** Mean time-course changes of the relative luminescence in HEK293T cells transfected with RXR-SmBiT and EcR-LgBiT reporters. Either an *EcI*-containing vector (EcI +) or an empty vector (Vector) was co-transfected, and the luminescence was monitored in real time in response to different doses of CF. Black triangles indicate the time point when CF was added to the medium. Data were normalized to the average of the basal luminescence reading immediately prior to treatment (dashed lines). R.L., relative luminescence. **(E,F)** Maximum relative luminescence in response to different doses of CF in the Vector **(E)** or EcI + **(F)** HEK293T cells. Bars represent means ± S.E.M. (*n* = 4). One-way ANOVA analysis identified a significant effect in the Vector cells [*F*_(7, 24)_ = 45.68, *p* < 0.0001, **(E)**] and in the EcI + cells [*F*_(7,24)_ = 112.9, *p* < 0.0001, **(F)**]. *, ***, and **** indicate *p* < 0.05, 0.001, and 0.0001, respectively, from multiple comparisons to the vehicle treatment using Dunnett’s correction. **(G,H)** 20E **(G)** and CF **(H)** dose-response relationship in the Vector and EcI + HEK293T cells. All values are the means ± S.E.M. (*n* = 4) replotted from panels **(B,C,E,F)**. Two-way ANOVAs identified a significant interaction of the 20E concentration with EcI expression [*F*_(7, 48)_ = 77.30, *p* < 0.0001, **(G)**] and only main effect of CF concentration independent of EcI expression [*F*_(7, 48)_ = 131.5, *p* < 0.0001, **(H)**]. **, and **** indicate *p* < 0.01, and 0.0001, respectively, from multiple comparisons using Sidak’s correction within each concentration of 20E between the EcI + and Vector cells.

Using the optimized EcR-RXR NanoBiT pair, we examined the dose dependence of the luciferase response to 20E in HEK293T cells co-transfected with an *EcI*-containing vector (EcI +). As an *EcI*-negative control, cells were transfected with an empty vector (Vector). An increase in relative luminescence upon 20E application was observed in the EcI + cells, while response to 20E was absent in the Vector cells (Figures 2A–C). In the EcI + cells, 20E-induced EcR-RXR dimerization was observed in a dose-dependent manner, with significant increases in relative luminescence exhibited in response to 20E concentrations of at least 300 nM compared to the vehicle treatment (*p* < 0.001 at 300 nM and *p* < 0.0001 at higher concentrations, *n* = 4, Figure 2C). The EC_50_ of the response in the EcI + cells was 2.20 μM 20E.

Chromafenozide is a non-steroidal EcR agonist, which is known to bind directly to *Drosophila* EcR and mimic 20E effect insects (Minakuchi et al., 2004). CF enters the cells through an EcI-independent pathway and activates EcR in a dose-dependent manner (Okamoto et al., 2018). We found that a dose-dependent response to CF was also observed in the NanoBiT assay, which occurred independently of the presence of EcI (Figures 2D–F). In both the Vector and EcI + cells, CF induced significant increase of relative luminescence at micromolar concentrations (Figures 2E,F). The EC_50_ of the response was 3.73 μM CF in EcI + and 4.76 μM CF in Vector cells.

Dose-response curves for 20E were significantly different between the Vector and EcI + cells at concentrations of at least 300 nM (Figure 2G), while those for CF were indistinguishable between the two cell types (Figure 2H). These results indicate that 20E but not CF requires EcI to enter HEK293T cells, consistent with the previous results using ecdysteroid-inducible gene expression system in HEK293 cells (Okamoto et al., 2018).

Taken together, the EcR-RXR NanoBiT assay can be effectively used to investigate ecdysteroid-importing function of EcI while using CF as an EcI-independent control.

### Establishment of a HEK293 Cell Line That Stably Expresses Ecdysone Importer

**FIGURE 3:**
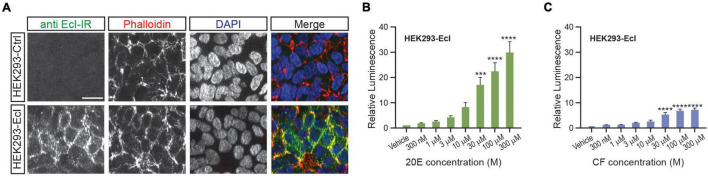
Establishment of an EcI-expressing HEK293 cell line. **(A)** Immunocytochemistry of HEK293-Ctrl and -EcI cells using anti-EcI antibodies (green in merged images). Filamentous actin of the cytoskeleton and cell nuclei were stained with phalloidin (red in merged images) and DAPI (blue in merged images), respectively. Scale bar, 20 μm. **(B,C)** Luciferase reporter activity in HEK293-EcI cells in response to different concentrations of 20E **(B)** or CF **(C)**. All values are the means ± S.E.M. [*n* = 6 in panel **(B)** and 5 in panel **(C)**]. One-way ANOVAs identified a significant effect of the 20E concentration [*F*_(7, 40)_ = 21.23, *p* < 0.0001, **(B)**] and the CF concentration [*F*_(7, 32)_ = 22.25, *p* < 0.0001, **(C)**]. ***, and **** indicate *p* < 0.001, and 0.0001, respectively, from multiple comparisons to the vehicle treatment using Dunnett’s correction.

As overexpression of EcI is necessary for 20E uptake into HEK293T cells, establishing a cell line that constitutively expresses EcI would be useful for reproducibly investigating EcI functions. We therefore designed and established a HEK293 cell line that stably expresses EcI (HEK293-EcI). The presence of *EcI* mRNA in the modified cells was first confirmed by RT-qPCR (Supplementary Table 1), and the expression and localization of EcI protein were examined by immunohistochemistry. HEK293-EcI cells exhibited robust anti-EcI-immunoreactivity (IR) signals, while the parental control HEK293 cells (HEK293-Ctrl) did not (Figure 3A). A phalloidin stain was included to mark filamentous actin, which visualizes the cytoskeletal structure. The anti-EcI-IR signals in HEK293-EcI cells were mostly localized on cell margins highlighted by phalloidin-positive regions, indicating that the EcI protein is primarily localized in membrane regions, with some localization observed throughout the HEK293-EcI cells (Figure 3A). This is a similar expression pattern to what has previously been observed for EcI in insect salivary gland and fat body (Okamoto et al., 2018). No toxicity due to EcI expression nor any phenotypic differences between the cell types was observed over the course of regular culture or experimentation on these cells.

To examine 20E responsiveness of HEK293-EcI cells, we performed an ecdysteroid-inducible luciferase reporter assay. After 24-h-treatment with 20E or CF, luminescent responses were observed in a dose-dependent manner in HEK293-EcI cells (Figures 3B,C). Notably, relative luminescence was constantly higher in 20E-treated cells as compared to CF-treated cells (Figures 3B,C), consistent with the previous study using HEK293 cells transiently transfected with an *EcI*-containing vector (Okamoto et al., 2018).

### Ecdysone Receptor NanoLuc Binary Technology Assay in HEK293-Ecdysone Importer and HEK293-Ctrl Cells

**FIGURE 4:**
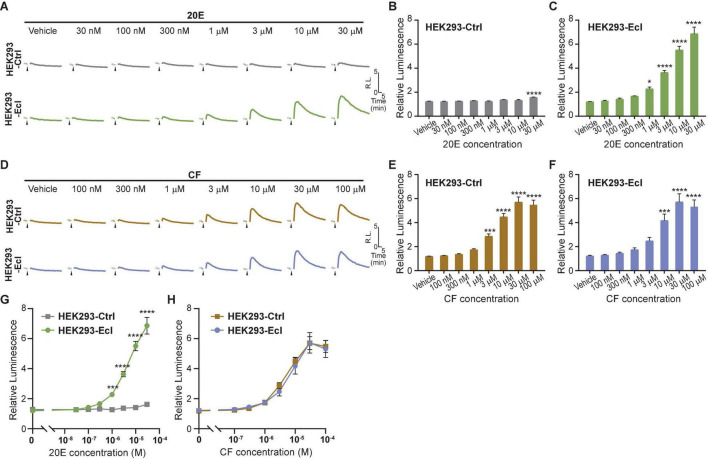
Detection of 20E and CF entry into HEK293-EcI and -Ctrl cells using the NanoBiT assay. **(A)** Mean time-course changes of the relative luminescence in HEK293-Ctrl or -EcI cells transfected with RXR-SmBiT and EcR-LgBiT reporters in response to different doses of 20E. **(B,C)** Maximum relative luminescence in response to different doses of 20E in HEK293-Ctrl **(B)** or -EcI **(C)** cells. Bars represent means ± S.E.M. (*n* = 4). One-way ANOVA analysis identified a significant effect in HEK293-Ctrl cells [*F*_(7, 24)_ = 7.11, *p* < 0.001, **(B)**] and HEK293-EcI cells [*F*_(7,24)_ = 79.05, *p* < 0.0001, **(C)**]. * and **** indicate *p* < 0.05, and 0.0001, respectively, from multiple comparisons to the vehicle treatment using Dunnett’s correction. Note that the larger variance across the samples in the HEK293-EcI cell data reduces the significances of the individual comparisons in a way which does not occur in the HEK293-Ctrl cells hence the smaller difference in panel **(B)** accounting for more significance in the multiple comparisons compared to that observed in panel **(C)**. **(D)** Mean time-course changes of the relative luminescence in HEK293-Ctrl or -EcI cells transfected with RXR-SmBiT and EcR-LgBiT reporters in response to different doses of CF. **(E,F)** Maximum relative luminescence in response to different doses of CF in the HEK293-Ctrl **(E)** or -EcI **(F)** cells. Bars represent means ± S.E.M. (*n* = 4). One-way ANOVA analysis identified a significant effect in HEK293-Ctrl cells [*F*_(7, 24)_ = 62.65, *p* < 0.0001, **(E)**] and HEK293-EcI cells [*F*_(7,24)_ = 23.29, *p* < 0.0001, **(F)**]. ***, and **** indicate *p* < 0.001, and 0.0001, respectively, from multiple comparisons to the vehicle treatment using Dunnett’s correction. **(G,H)** 20E **(G)** and CF **(H)** dose-response relationship in HEK293-Ctrl and -EcI cells. All values are the means ± S.E.M. (*n* = 4) replotted from panels **(B,C,E,F)**. Two-way ANOVAs identified a significant interaction of the 20E concentration with EcI expression [*F*_(7, 48)_ = 69.72, *p* < 0.0001, **(G)**] and only a main effect of CF concentration, independent of EcI expression [*F*_(7, 48)_ = 68.88, *p* < 0.0001, **(H)**]. ***, and **** indicate *p* < 0.001, and 0.0001, respectively, from multiple comparisons using Sidak’s correction within each concentration of 20E between HEK293-Ctrl and HEK293-EcI cells.

To maximize efficiency and reproducibility of the assay system to examine EcI-dependent 20E uptake, we lastly conducted the EcR-RXR NanoBiT assay in HEK293-EcI cells. We transfected HEK293-EcI or -Ctrl cells with EcR-LgBiT and RXR-SmBiT and monitored luciferase activity upon 20E or CF treatment. As expected, 20E induced larger increase of luminescence in HEK293-EcI cells compared to HEK293-Ctrl cells (Figure 4A). Interestingly, at the highest concentration tested (30 μM), a significant increase of relative luminescence compared to the vehicle control was observed in HEK293-Ctrl cells (*p* < 0.0001, *n* = 4, Figure 4B). In HEK293-EcI cells, 20E induced EcR-RXR NanoBiT luminescence in a dose-dependent manner, which exhibited significant responses compared to the vehicle control at concentrations of at least 1 μM (*p* < 0.05, *n* = 4, Figure 4C). We did not observe the HEK293-EcI response to have reached a clear peak plateau in those dose series used here. This precluded identification of the EC_50_ of the response to 20E in this system. CF induced EcR-NanoBiT luminescence in both HEK293-EcI and -Ctrl cells in a dose-dependent manner (Figures 4D–F). The EC_50_ of the response was 6.25 μM CF in HEK293-EcI and 4.93 μM CF in HEK293-Ctrl cells.

Dose-response curves for 20E were significantly different between HEK293-EcI and HEK293-Ctrl cells at concentrations of at least 1 μM (*p* < 0.001, *n* = 4, Figure 4G). Although 30 μM 20E application induced a slight increase of luminescence compared to the vehicle control in HEK293-Ctrl cells, the maximum relative luminescence induced by the same concentration of 20E was more than fivefold higher in HEK293-EcI cells (*p* < 0.0001, *n* = 4, Figure 4G). As expected, dose-response curves for CF were indistinguishable between the two cell lines (Figure 4H). Importantly, the magnitudes of relative luminescence induced by 20E and CF were in the same order (6.9 and 5.7 in response to 30 μM 20E and CF, respectively, in HEK293-EcI cells), suggesting that CF response can be detected without the potential side effects observed in the ecdysteroid-inducible luciferase reporter assay (Figure 3C).

In summary, by combining the EcR-RXR NanoBiT assay and HEK293-EcI cells, we successfully established a method that can rapidly monitor ecdysteroid entry into cultured cells. This method is expected to facilitate the future effort to screen and identify chemical compounds that can either inhibit or facilitate EcI-mediated transport of ecdysteroids across cellular membranes.

## Discussion

In the current study, we developed a novel assay system for detecting cellular uptake of ecdysteroids in a heterologous system by combining the EcR-RXR NanoBiT assay and HEK293-EcI cells. The EcR-RXR NanoBiT assay monitors formation of the EcR-RXR heterodimer complex upon 20E binding in real time, making it a reliable tool to assess the incorporation of steroid hormone into animal cells as mediated by EcI.

Upon optimization of NanoBiT pairs as an ecdysteroid sensor, we found that EcR-USP NanoBiT was unsuitable to detect a response to 20E due to no increase in luminescence (Figure 1C). This was likely due to the successful NanoBiT pairs (all except 6 and 8) presenting high background luminescence even prior to 20E treatment, which did not increase luminescence substantially from the baselines (Supplementary Figure 2). This suggests that, consistent with previous findings (Palli et al., 2003, 2005), EcR-USP interaction in HEK293 cells occurs even in the absence of 20E, making it difficult to use this nuclear receptor pair for rapidly monitoring 20E entry into the cells.

Among the eight EcR-RXR combinations investigated, we found that the two combinations in which both EcR and RXR have a C-terminal tag induced the highest relative luminescence (Figure 1E, pairs 4 and 8), suggesting that the two subunits fused to the C-termini may be able to more readily associate with each other when RXR and EcR dimerize. This is consistent with the previous finding of functional domains of EcR and RXR. The N-terminal regions of both EcR and RXR are close to the Zn-1 module which forms part of the dimer interface, while the C-terminal regions of both proteins have been found not to contribute to the dimer interface (Devarakonda et al., 2003).

The biggest advantage of this NanoBiT system is that cellular incorporation of EcR agonists can be detected without discernible side effects. This was evident by comparing EcR responses to 20E and CF in the traditional luciferase reporter assay and NanoBiT assay in HEK293-EcI cells. The relative responses to 20E and CF exhibited more than threefold difference in the ecdysteroid-inducible luciferase reporter assay (Figures 3B,C) in a way which was consistent with previous findings (Okamoto et al., 2018). On the other hand, responses to 20E and CF in the NanoBiT assay were mostly comparable (Figures 4C,F), indicating that the induction of dimerization by 20E and CF occurs and decays in a similar manner which should lead to similar expression of genes under the EcREs. This suggests that unknown chronic effects induced by long term (24-h) incubation with 20E or CF were responsible for the differing responses in the traditional assay. The more comparable consistency between the CF and 20E response in the EcR-RXR NanoBiT system indicates that the assay can monitor EcR ligand entry into the cells more directly, excluding unwanted side effects. This feature will be critically important in our future effort to screen for chemical reagents that affect cellular uptake of steroid hormones, as use of CF as a control for EcI will require the response to be as similar as possible in all aspects, excluding EcI dependence. Moreover, as the NanoBiT system can be used to analyze temporal dynamics of ligand-dependent EcR-RXR heterodimer formation, it may be useful for further investigating modes-of-action of investigated chemical reagents.

We initially anticipated that the NanoBiT assay in HEK293 cells expressing EcI would improve sensitivity of the EcR response to 20E. The threshold concentration of the EcR response to 20E in mammalian cells is ∼3 μM in the traditional luciferase reporter assay, which is ∼30 times higher than that in insect cells [<100 nM; (Yao et al., 1992; Okamoto et al., 2018)]. We found that sensitivity of the NanoBiT assay was slightly higher than that of the traditional assay in mammalian cells, with the threshold concentration of the EcR response to 20E in EcI + cells being 300 nM (Figure 2C). Constitutive expression of EcI did not improve the sensitivity of the assay, with the threshold concentration in HEK293-EcI cells being 1 μM (Figure 4C). Based on these results, we assume that the difference of the EcR sensitivity to 20E between insect and mammalian cells is mainly derived from the dimerizing partners: endogenous USP in insect cells and RXR in mammalian cells. Indeed, it has been shown that ecdysteroids can bind to EcR more efficiently in the *Drosophila* EcR-USP complex than in the EcR-RXR complex (Yao et al., 1993). However, the EcR-USP NanoBiT assay did not work in our study, likely due to the ligand-independent dimerization of EcR-USP in HEK293T cells (Figure 1). *USP* genes in dipterans and lepidopterans (including *D. melanogaster USP* used in this study) are evolutionally diverged, while *RXR/USP* gene in other arthropods, including coleopterans and hymenopterans, are phylogenetically closer to the vertebrate *RXR* (Maestro et al., 2005). Therefore, utilization of RXR/USP from coleopterans and hymenopterans such as the cockroach *Blattella germanica* as the dimerization partner of the *Drosophila* EcR might improve the sensitivity, as well as dynamic range, of this NanoBiT sensor.

Although it is not our current focus, NanoBiT ecdysteroid sensor can be utilized in insect cell lines, such as *Drosophila* S2 cells, or cells from other insect species, to further investigate detailed molecular and cellular mechanisms of ecdysteroid incorporation in various insect species. An *EcI* knockout S2 cell line established in the previous study (Okamoto et al., 2018) may be useful as the negative control in such studies.

In summary, we developed a real time monitoring system and a stable cell line to rapidly detect insect steroid hormone entry into cultured cells. This system can be used as a complementary tool to insect-based models, which will aid our future effort to develop novel pharmacological tools to manipulate insect steroid hormone signaling. Further study on EcI functions is expected to deepen our understanding of steroid hormone biology, which may have a far-reaching impact beyond arthropod research.

## Data Availability Statement

The original contributions presented in the study are included in the article/Supplementary Material, further inquiries can be directed to the corresponding author.

## Author Contributions

NY and SH-Y: conceptualization, supervision, and funding acquisition. MM, RB, HC, NY, and SH-Y: methodology and investigation. MM, RB, and SH-Y: writing–original draft. MM, NY, and SH-Y: writing–review and editing. All authors contributed to the article and approved the submitted version.

## Conflict of Interest

RB, SH-Y, and NY have a patent (U.S. Patent No. 10,228,380) relevant to this work. The remaining authors declare that the research was conducted in the absence of any commercial or financial relationships that could be construed as a potential conflict of interest.

## Publisher’s Note

All claims expressed in this article are solely those of the authors and do not necessarily represent those of their affiliated organizations, or those of the publisher, the editors and the reviewers. Any product that may be evaluated in this article, or claim that may be made by its manufacturer, is not guaranteed or endorsed by the publisher.

## Supplementary Material

Supplementary Figure 1 Regions within the expressed sequence from the NanoBiT plasmids. Regions are to scale based on the inserted gene being RXR. Start and stop codons are presented in green and red, respectively. Restriction/ligation sites are marked above the regions.

Supplementary Figure 2 Time course of raw luminescence reads in HEK293T cells expressing EcI and each of the NanoBiT EcR-USP (left) or EcR-RXR (right) combinations 1–8 listed in Figure 1B. Figures 1C,D present the normalized data shown here. Dashed lines indicate the time point when either EtOH or 10 μM 20E was added to the medium. RLU, relative light units.

Supplementary Table 1 Gene expression of EcI (OATP74D) in the modified HEK293 cells. The table contains the RQ, Mean C_T_, ΔC_T_, and ΔΔC_T_ values for the gene expression analysis. The assay was run in triplicates of each sample. The table shows the average of triplicates. RQ values from targets with undetermined Mean C_T_ were approximated by assuming *C*_T_ = 40.000.
