# Supplementary material for: Rapid Assessment of Insect Steroid Hormone Entry Into Cultured Cells
Source: Front Physiol. 2022 Jan 26;12:816058. doi: 10.3389/fphys.2021.816058 (PMC8824665; doi:10.3389/fphys.2021.816058)
Supplement: Supplementary Figure 1 — Regions within the expressed sequence from the NanoBiT plasmids. Regions are to scale based on the inserted gene being RXR. Start and stop codons are presented in green and red, respectively. Restriction/ligation sites are marked above the regions. [file Image_1.pdf]

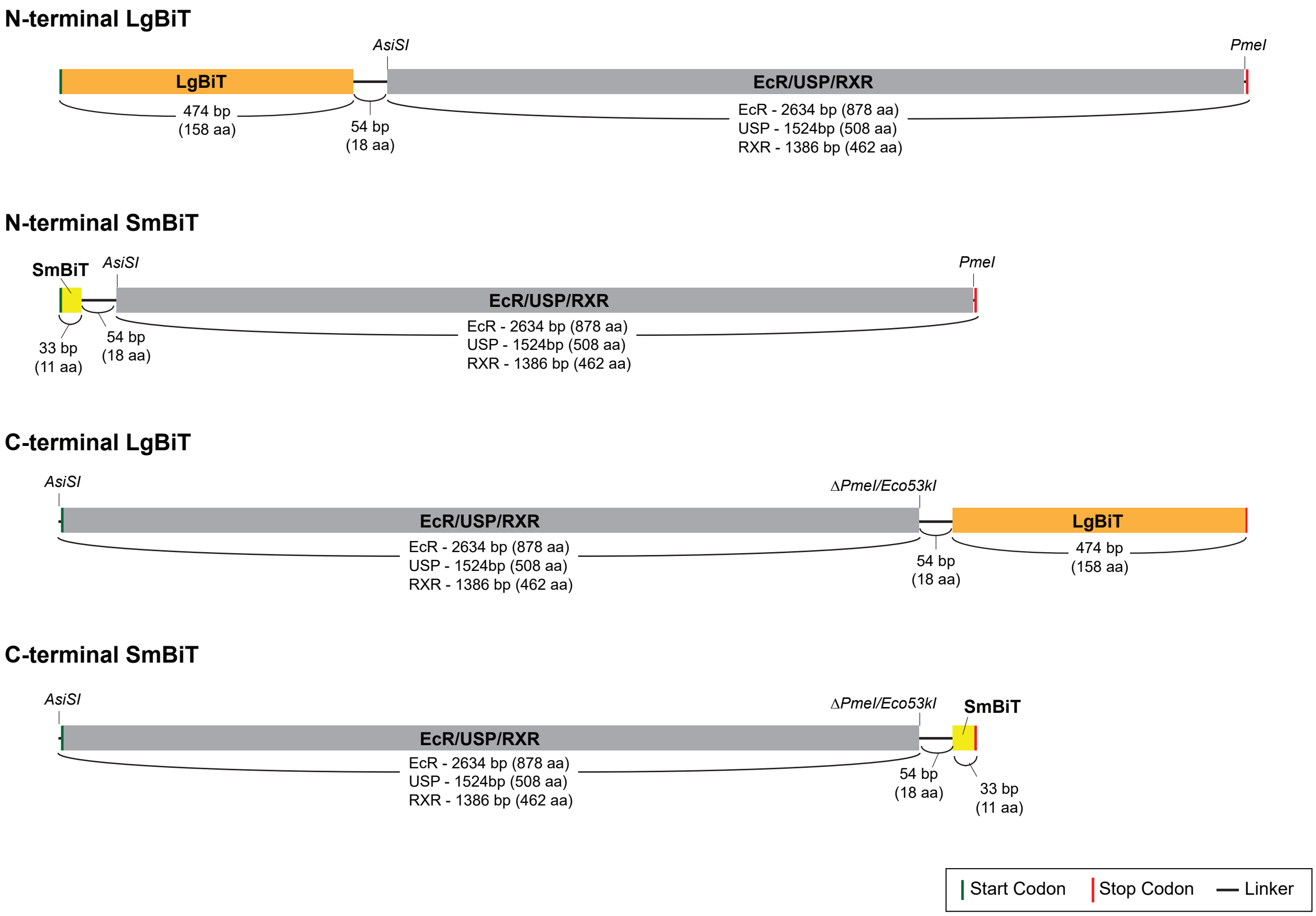

Supplementary Figure 1 Masterson et al.
