# Supplementary figures and images for: Rapid Assessment of Insect Steroid Hormone Entry Into Cultured Cells
Source: Front Physiol. 2022 Jan 26;12:816058. doi: 10.3389/fphys.2021.816058 (PMC8824665; doi:10.3389/fphys.2021.816058)

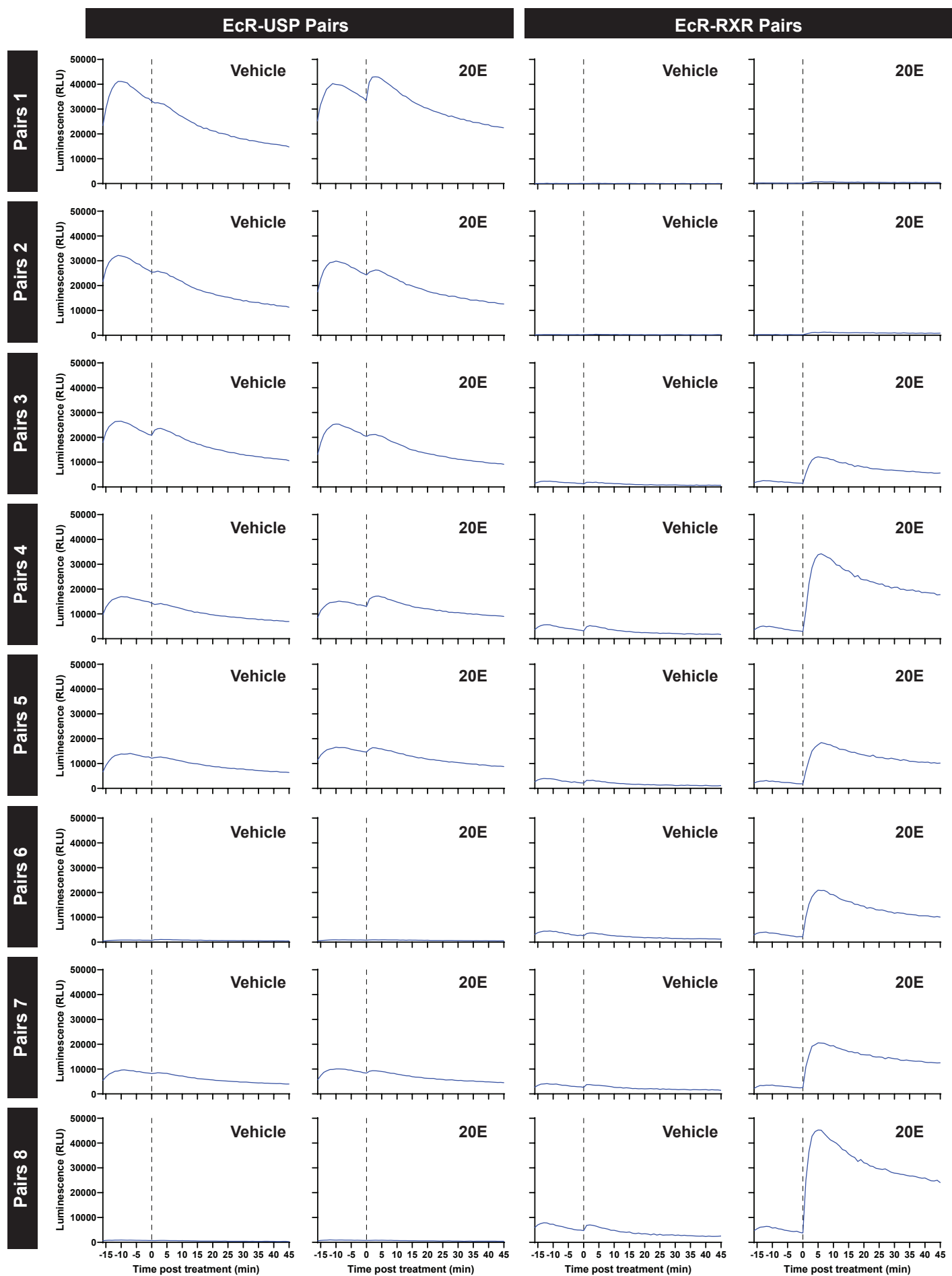

Supplementary Figure 2 Masterson et al.

Supplement: Supplementary Figure 2 — Time course of raw luminescence reads in HEK293T cells expressing EcI and each of the NanoBiT EcR-USP (left) or EcR-RXR (right) combinations 1–8 listed in Figure 1B. Figures 1C,D present the normalized data shown here. Dashed lines indicate the time point when either EtOH or 10 μM 20E was added to the medium. RLU, relative light units. [file Image_2.pdf]
