# Supplementary material for: Rapid Assessment of Insect Steroid Hormone Entry Into Cultured Cells
Source: Front Physiol. 2022 Jan 26;12:816058. doi: 10.3389/fphys.2021.816058 (PMC8824665; doi:10.3389/fphys.2021.816058)
Supplement: Supplementary Table 1 — Gene expression of EcI (OATP74D) in the modified HEK293 cells. The table contains the RQ, Mean CT, ΔCT, and ΔΔCT values for the gene expression analysis. The assay was run in triplicates of each sample. The table shows the average of triplicates. RQ values from targets with undetermined Mean CT were approximated by assuming CT = 40.000. [file Table_1.pdf]

Cell line	Gene target	Ct Mean	Δ Ct Mean	Δ Ct SE	Δ Δ Ct Mean	RQ
HEK293-EcI	EcI/OATP64D	23.944	0.918	0.559	-15.516	46853
	Beta-Actin	23.026				
HEK293-Ctrl	EcI/OATP64D	Undetermined	16.433		0	1
	Beta-Actin	23.567				

Supplementary Table 1 Masterson et al.
